# Integrating Adjuvant HPV Vaccination into Cervical Dysplasia Management After LLETZ/Conization

**DOI:** 10.3390/jcm15093424

**Published:** 2026-04-30

**Authors:** Ecaterina Tomaziu-Todosia Anton, Daniela Roxana Matasariu, Alexandra Ursache, Gabriel-Ioan Anton, Raluca Anca Balan, Ramona-Gabriela Ursu, Ioana-Sadiye Scripcariu, Alexandru Cărăuleanu, Carmen Pintilescu, Demetra Gabriela Socolov

**Affiliations:** 1 Grigore T. Popa University of Medicine and Pharmacy Iasi, 700115 Iasi, Romania; tomaziu-todosia.ecaterina@d.umfiasi.ro (E.T.-T.A.); alexandra.ursache@umfiasi.ro (A.U.); antongabrielioan@gmail.com (G.-I.A.); raluca.balan@umfiasi.ro (R.A.B.); ramona.ursu@umfiasi.ro (R.-G.U.); ioana.scripcariu@umfiasi.ro (I.-S.S.); ale.carauleanu@umfiasi.ro (A.C.); demetra.socolov@umfiasi.ro (D.G.S.); 2 Department of Obstetrics and Gynecology, Cuza Voda Hospital, 700038 Iasi, Romania; 3 “Elena Doamna” Clinical Hospital of Obstetrics and Gynecology, 700398 Iasi, Romania; 4 Faculty of Economics and Business Administration, Alexandru Ioan Cuza University of Iasi, 700505 Iasi, Romania

**Keywords:** HPV, cervical dysplasia, persistence, vaccination, nonavalent Gardasil^®^ vaccine, cervical intraepithelial neoplasia, conization, LLETZ

## Abstract

**Background:** In this study, we evaluated the effects of the nonavalent Gardasil^®^ vaccine in a heterogeneous cohort of women who underwent conization or LLETZ for various degrees of CIN, aiming to assess the adjuvant effect of vaccination on HPV clearance. **Methods:** We conducted a three-year prospective study with a two-year follow-up of 219 patients presenting to our facility for cervical dysplasia. All patients underwent HPV genotyping and were divided into HPV-vaccinated and non-vaccinated groups. **Results:** We detected a significant association between the final outcome and vaccination timing (*p*-value = 0.005). All women vaccinated before conization achieved viral clearance; 94.9% of those vaccinated at the time of conization/LLETZ and 68.6% of those vaccinated after the excisional procedure became HPV-negative. Logistic regression showed that, with increasing age, the likelihood of healing after vaccination decreased by approximately 9% per additional year, and non-16/18 HPV-positive women had a 5.5-fold higher chance of healing after vaccination compared with those HPV-positive for 16 and 18 genotypes. **Conclusions:** Adjuvant prophylactic HPV vaccination in the context of surgical treatment for cervical precancerous lesions is significantly associated with a reduced risk of lesion recurrence and HPV persistence/reinfection when administered prior to or at the same time as the excisional procedures.

## 1. Introduction

Cervical cancer remains one of the most preventable malignancies, being almost exclusively linked to persistent infection with oncogenic types of human papillomavirus (HPV), especially high-risk (hr) types [1,2,3,4,5,6]. The introduction of prophylactic HPV vaccines marked a significant public health achievement in primary prevention, with three types having become available over time: the bivalent Cervarix vaccine, the quadrivalent Gardasil^®^ vaccine, and the nonavalent Gardasil^®^ 9. Guidelines recommend vaccination before the onset of sexual activity for optimal protection, and research has confirmed its effectiveness in reducing HPV infection and subsequently decreasing both CIN lesions and invasive cervical cancer in vaccinated populations [7,8,9,10,11,12,13,14].

But in recent years, interest has grown in the adjuvant use of HPV vaccination after excisional treatments, with a particular focus on high-grade CIN 2 and 3 lesions. Despite treatment, the risk of persistent or recurrent disease remains substantial, ranging from 5–10% to 30–40% at 12–24 months, largely attributable to persistent HPV infection or reinfection with hr genotypes [12,15,16,17,18,19]. Meta-analyses have shown a significant reduction in the recurrence of CIN2+ lesions among vaccinated women compared with unvaccinated women, with odds ratios of 0.35–0.51, corresponding to a 49–65% relative reduction in recurrence. This protective effect has been documented for both bivalent and quadrivalent vaccines [7,9,10,20,21,22,23]. Recent evidence also suggests strong adjuvant potential for the nonavalent vaccine [17,24].

However, the mechanism underlying this adjuvant effect remains incompletely understood. Proposed explanations include the neutralizing effect of antibodies produced secondary to vaccination, which prevent reinfection or reactivation of latent HPV; cross-protection against non-vaccine types; and improvement in the local immune microenvironment following excisional surgery, creating protective, vaccine-induced local conditions [8,16,18,22,25,26]. Another hypothesis is that surgical excision through conization or LLETZ may reduce local inflammation, thereby restoring a non-HPV cervical environment in which vaccination could prevent reinfection or the persistence of viral particles [13,16,23].

Despite encouraging data from retrospective cohorts, prospective studies, and meta-analysis, research remains controversial [10,16,22,25]. Some studies demonstrate a significant reduction of up to 80% in recurrence rates among vaccinated women [10,16,20,22], while others find no statistically significant benefit [25]. Variations in vaccine timing (before the surgical intervention or after it), population heterogeneity, follow-up time frame, the predominance of retrospective studies, and lack of randomized controlled trials contribute to these discrepancies. Therefore, while HPV vaccination is firmly established as the basis for cervical cancer prevention, its adjuvant intervention in the management of CIN lesions remains debatable, as does its optimal administration timing to maximize benefits [10,16,20,22,25].

Most existing studies have focused exclusively on women with high-grade lesions, whereas data regarding the adjuvant benefit of vaccination in patients with low-grade lesions remain scarce. This selection bias toward homogenous CIN2+ cohorts may overestimate the vaccine’s efficacy by excluding women with less severe or self-limited disease, who may have different natural histories and immune responses [7,9,10,12,15,16,17,18,19,20,21,22,23]. Consequently, the widespread relevance of these findings to the broader population of women undergoing excisional procedures for any grade of CIN remains limited [27,28].

Thus, we decided to evaluate the effects of the nonavalent Gardasil^®^ vaccine administered to an inhomogeneous group of women, who underwent conization or LLETZ for various degrees of CIN, in a country where the HPV vaccination program was not fully implemented until 2019 (with the first program in 2008 failing to gain acceptance by the population). Our prospective research, including all grades of CIN, might prove valuable, improving comprehension and global understanding of the true adjuvant effects of HPV vaccination. Moreover, prospective evaluation across diverse CIN categories could clarify the protective role of HPV vaccination in preventing recurrence and guide the evidence-based implementation of adjuvant vaccination strategies to improve outcomes, depending on the CIN lesion type. As a secondary objective, we aimed to identify potential factors associated with the risk of recurrent cervical dysplasia among vaccinated women—age, degree of dysplasia at the moment of surgical treatment, timing of vaccination, and HPV genotype.

## 2. Materials and Methods

Our prospective study was conducted in a private clinic in North-East Romania. The time frame extended from May 2022 to May 2025 with a two-year follow-up period. Each patient enrolled in this study signed their written informed consent, approved by the Ethics Committee of Avicena Clinic Iasi (No. 2/10.01.2022).

**Figure 1:**
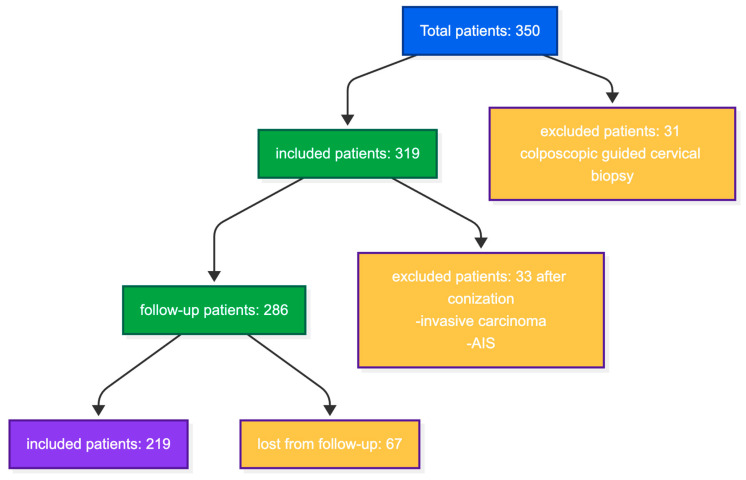
Flow chart of our inclusion process.

We included all women who presented to our facility for cervical dysplasia, provided informed consent, were older than 18 years, and underwent a surgical excisional procedure (LLETZ or electrosurgical conization) for their lesions, with histologically negative margins after the procedure (Figure 1). We recorded the following variables for each included woman: age, parity, smoking status, age at first intercourse, number of sexual partners, education level, HPV vaccination history and timing, histopathological evaluation with CIN grading, margin status, and colposcopy findings.

The exclusion criteria were as follows: age under 18 years, consent withdrawal, having undergone an ablative procedure, microinvasion detected after the histopathological evaluation, invasive cervical cancer, loss of follow-up, impossibility of recording vaccination status, glandular lesions, ongoing pregnancy, and a history of hysterectomy. We also excluded all women who might have impaired immunity, such as women living with HIV, women with hepatitis B or C, women with autoimmune diseases, and those with any other malignancies present (Figure 1).

Outcome assessment was performed at the end of the follow-up period with knowledge of the vaccination status, as some patients received the HPV vaccine after conization.

Although conducted prospectively, this study was not registered in a public clinical trial database because it was designed as an observational, investigator-initiated cohort study rather than a randomized trial in a private outpatient clinic.

### 2.1. Liquid-Based Cytology and HPV Testing

Cervical samples were collected using a cyto-brush and stored in a PreservCyt solution (Hologic, Marlborough, MA, USA) for liquid-based cytology and HPV testing. A ThinPrep T2000 slide processor (Hologic) was used to prepare thin-layer cytology slides that were stained using the Papanicolaou method. The cytology slides were then evaluated using the Bethesda system.

Human papillomavirus detection and genotyping were performed on all samples using the Allplex™ HPV28 Detection kit (Seegene Technologies Inc. Europe, Düsseldorf, Germany) according to the manufacturer’s instructions. The kit is a multiplex real-time PCR assay that enables simultaneous amplification and detection of target nucleic acids from 19 hrHPV types (16, 18, 26, 31, 33, 35, 39, 45, 51, 52, 53, 56, 58, 59, 66, 68, 69, 73, 82) and 9 low-risk HPV (lrHPV) types (6, 11, 40, 42, 43, 44, 54, 61, 70), as well as an internal control (IC).

### 2.2. Cervical Conization/LLETZ and Histopathological Assessment

The criteria for LLETZ/conization were as follows: CIN2+ diagnosis via colposcopy-directed biopsy and/or endocervical curettage; repeated HSIL cytology in at least two Pap smears at six-month intervals in patients with histological low-grade CIN1 lesions or no lesion, after excluding vaginal HSIL; and cases with persistent CIN1 or HPV-positive ASCUS/LSIL extending beyond two years of surveillance, particularly when accompanied by additional patient-specific factors such as sustained hrHPV infection, elevated patient anxiety (such as family history of cervical cancer and at in young best friends), or significant barriers to ensuring adequate and reliable long-term follow-up. All women were tested for HPV before conization/LLETZ, and their HPV types were classified as hrHPV according to the International Agency for Research on Cancer (IARC) classification [29].

The same expert performed all cervical interventions and colposcopic examinations. The interventions were performed in our outpatient facility, under intravenous anesthesia with pethidine chlorhydrate (Mialgin^®^, Biogroup, Levallois-Perret, France) and local anesthesia with xylene. Additionally, procedures were performed under colposcopic guidance, using LLETZ/electrosurgical conization techniques.

Colposcopic examinations were interpreted using the Swede Colposcopic Index, which incorporates assessments of lesion coloration, margins, and surface characteristics, vascular patterns, and iodine staining. A total of 1 mL of 1% Xylocaine was administered into each cervical quadrant for local anesthesia, and LLETZ was performed under colposcopic guidance, with the loop size selected based on the extent of the lesion. In cases of suspected endocervical involvement, an additional targeted endocervical excision was performed using a smaller loop. When the exocervical lesion exceeded the capacity of a single excision, two or more systematic sweeps were carried out. Hemostasis was achieved by selective coagulation using needle diathermy.

Excisional specimens were anatomically oriented and fixed in 10% neutral buffered formalin. Each specimen was entirely processed and embedded into 3 to 14 paraffin blocks (median: 6), and two anatomopathologists evaluated each specimen to ensure accuracy.

### 2.3. Nonavalent Gardasil^®^ Vaccination

Vaccination with the nonavalent anti-HPV vaccine was proposed to every woman scheduled for LLETZ/conization with no history of prior anti-HPV vaccination. All the women included were informed of the possible risks and benefits of HPV vaccination. Post-conization follow-up controls were scheduled at three months, six months, one year, and two years after excision. The evaluation included a Pap smear, HPV testing, and a colposcopy. None of the patients underwent previous vaccination with either of the three prophylactic anti-HPV vaccines: Cervarix^®^ (GlaxoSmithKline (GSK), London, UK), Gardasil^®^ (Merck & Co., Rahway, NJ, USA), tetravalent, or Gardasil^®^, nonavalent.

The vaccination was recommended at the time the conization/LLETZ was scheduled. The cytologists who reviewed the Pap smear samples and the laboratory that performed HPV genotyping were unaware of the vaccination status of the women included in our study.

### 2.4. Persistent/Recurrent Disease

Persistent disease was defined as the continued presence of cytological abnormalities of any grade or HPV-positive results detected at consecutive follow-up evaluations after primary excisional treatment with or without vaccination, with no documented negative Pap smears in between. Recurrent disease was defined as the reappearance of cytological abnormalities (ASCUS+) after at least one documented negative post-treatment follow-up, regardless of HPV status.

### 2.5. Viral Clearance

Viral clearance was considered after at least one documented negative post-treatment HPV follow-up for the initial HPV genotype/s present.

### 2.6. Vaccination Moment

The pre-conization/LLETZ vaccination group consists of women who received the last Gardasil dose at least one month before the excisional procedure. The simultaneous vaccination with the excisional procedure group included women who initiated the vaccination schedule, with the first dose administered within 1 month after the conization. The post-conization vaccinated group consisted of women who started their vaccination, with the first dose administered more than 6 months after the excisional procedure.

### 2.7. Statistical Analysis

We performed statistical analyses using the Statistical Package for the Social Sciences (SPSS), version 25.0 (IBM, Armonk, NY, USA). We described the dataset by presenting absolute and relative frequencies for categorical variables and by calculating the mean and standard deviation for continuous variables. To assess statistically significant differences between categorical variables, we applied the Chi-squared test or Fisher’s exact test, using a two-tailed approach. The level of significance was set at 0.05. To assess differences between the outcome and cytology results at the six-month, one-year, and two-year follow-up points, we applied Fisher’s exact test and, for better visualization of the data, used a heatmap to represent the magnitude of individual values within our dataset as color. Proportions were calculated as the percentage of women in each group with the indicated cytology results at each time point of our follow-up period—six months, one year, and two years. The more intense the color, the higher the value for the frequency of the data. To assess the association between clinical predictors and treatment outcome, a multinomial logistic regression model was estimated. The overall significance of each predictor was determined by likelihood ratio tests comparing the full model with a reduced one excluding the variable of interest. Model performance was quantified using Cox and Snell’s pseudo R^2^ (0.095) and Nagelkerke’s pseudo R^2^ (0.131).

## 3. Results

From June 2020 to May 2025, 350 women were examined in our center for cytology-detected cervical dysplasia. In total, 31 of them were excluded from our analysis after the colposcopic examination, and all of them only had a biopsy performed.

After applying the inclusion and exclusion criteria, we restricted our analysis to 286 patients undergoing only conization/LLETZ. An additional 67 women were excluded due to being lost to follow-up after conization/LLETZ, with 219 remaining for the analysis (80 vaccinated and 139 unvaccinated). The selection process is depicted in the flow chart in Figure 1.

When analyzing smoking habits, we did not detect any statistically significant difference in outcomes between our two groups of vaccinated and unvaccinated women (*p* = 0.545). Of the 219 women included, 142 were current smokers, and 77 were non-smokers for at least 5 years prior to study enrollment (our evaluation form did not include previous smoking habits). The median age at first sexual intercourse was 18 years, with no differences between the two groups (*p* = 0.269), and the median number of sexual partners was three, with no statistically significant difference found between the groups (*p* = 0.533).

The level of education between the two groups did not show a statistically significant difference (*p* = 0.673). Of the 219 women included, 98 had completed higher education, and 121 had completed secondary education. Regarding parity, 100 patients were nulliparous, and 119 had one or more deliveries. No statistically significant difference was observed between the vaccinated and unvaccinated groups (*p* = 0.086).

**Figure 2:**
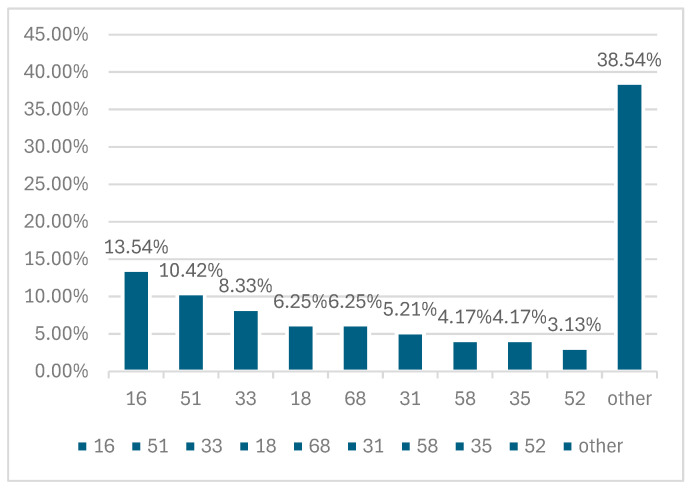
HPV genotype prevalence among the 219 women included in our study.

**Figure 3:**
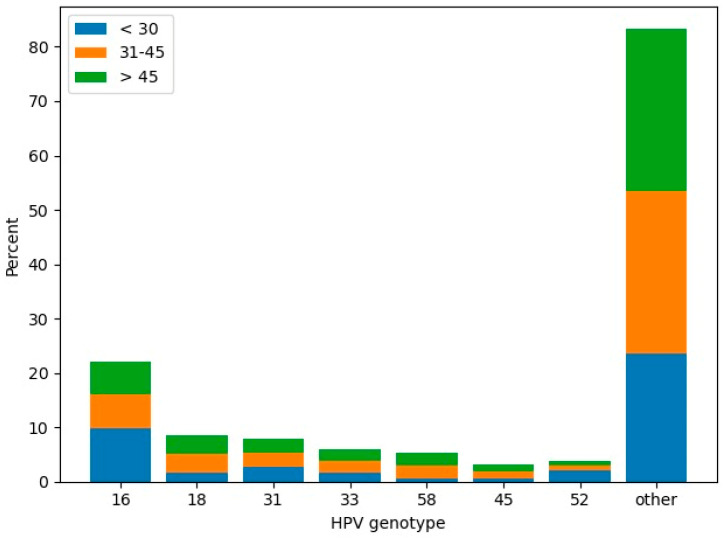
Age-specific prevalence of HPV genotypes among the women included in our study.

**Figure 4:**
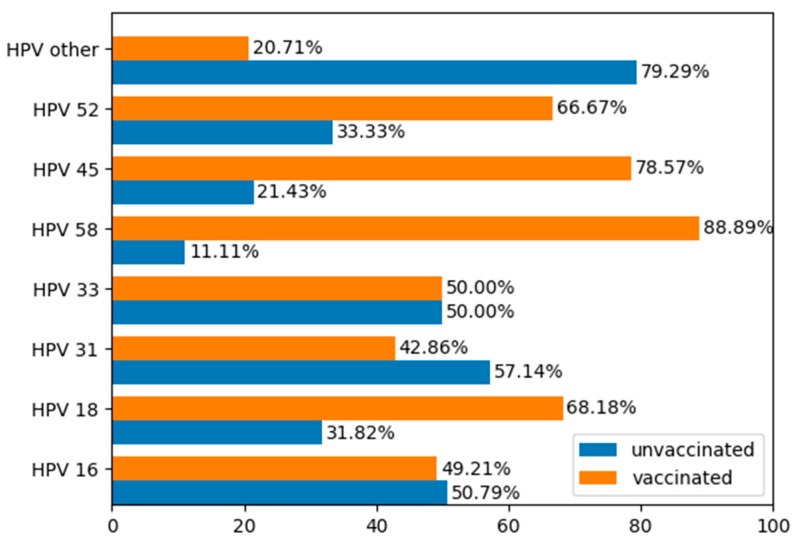
HPV genotype prevalence in the two groups in our study (HPV-vaccinated and unvaccinated women).

hrHPV was detected in 186 women, and 33 of them proved to be HPV-negative. Data regarding the HPV genotypes present in our population is illustrated in Figure 2, Figure 3 and Figure 4.

The vaccination status of patients who had undergone conization indicated that 80 women (36.53%) accepted vaccination at the time of conization and were enrolled in the vaccinated group, whereas 139 women (63.47%) refused vaccination at the time of conization and constituted the non-vaccinated group. Of the 33 HPV-negative women included in our study, 9 desired vaccination, and 24 refused it.

**Figure 5:**
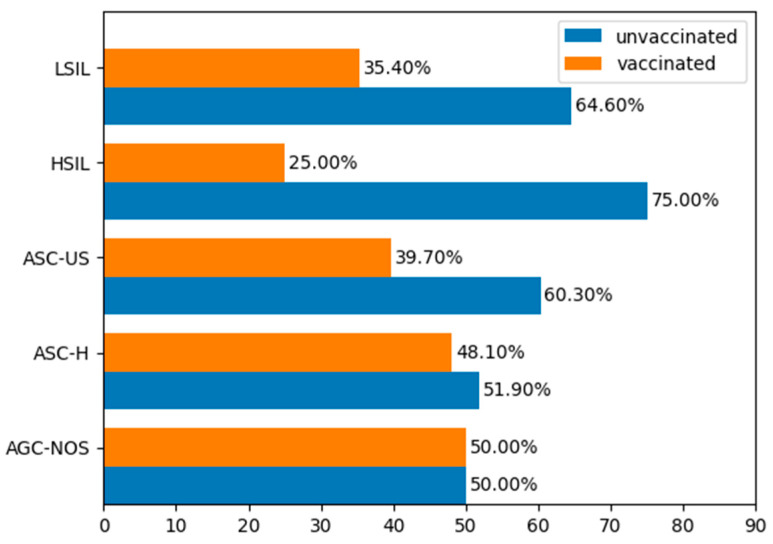
Pap smear status in the study groups before conization.

The Pap smear results before vaccination in both the vaccinated and the unvaccinated groups are presented in Figure 5.

After pathological examination of the specimens, we divided our patients by CIN1 and CIN2+ lesions. Flowcharts summarizing the characteristics of the 219 women included in our study are presented in the Supplementary Material section (Pap smear results, HPV status, vaccination status, and follow-up outcomes). Post-conization follow-up controls were scheduled at three months, six months, one year, and two years after the excisional procedure, involving a Pap smear, HPV testing, and a colposcopy.

There were no significant differences in the final outcome by vaccination status (*p*-value of 0.845), indicating that neither persistence nor viral clearance was associated with vaccination status (83.8% of vaccinated women and 85.6% of unvaccinated women achieved viral clearance). A separate analysis of HPV-positive cases alone did not significantly affect our results (*p*-value of 0.51). However, we detected a significant association between the final outcome and the timing of vaccination (*p*-value of 0.005). This suggests that HPV persistence and clearance are associated with the timing of vaccination. All women vaccinated before conization achieved viral clearance; 94.9% of those vaccinated at the time of conization/LLETZ and 68.6% of those vaccinated after the excisional procedure became HPV-negative (Table 1).

**Figure 6:**
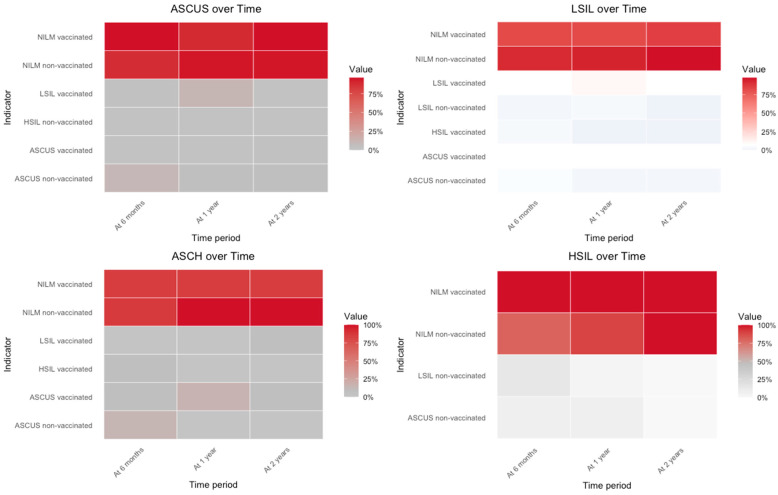
Heatmap showing the proportion of our cytology results at six-month, one-year, and two-year follow-up points, stratified by HPV vaccination status (in our analysis, red denotes the highest intensity).

Statistically significant differences were observed between the outcome and cytology results during the six-month, one-year, and two-year follow-up periods among vaccinated women, with *p* values of 0.002, <0.0001, and 0.001, respectively, as shown in Figure 6 and detailed in Table 2. Among HPV-vaccinated women, the association was statistically significant at the 1-year follow-up using a 1% threshold (*p* < 0.0001) and at the 6-month control point using a 5% threshold (*p* = 0.014). The association at the 2-year follow-up was not statistically significant (*p* = 0.054). For the CIN 1 category: 30 women (81.1%) experienced viral clearance after conization; 1 woman (2.7%) had viral clearance after conization and vaccination; and 6 cases (16.2%) remained HPV positive. For the CIN 2+ cases: 142 women (78%) achieved viral clearance after conization alone; 13 women (7.1%) became HPV negative after conization and vaccination; and 27 cases (14.8%) remained HPV positive.

11 women chose vaccination after the 6-month follow-up, when they were detected to be HPV positive. Among these 11 women who were vaccinated within 6 months of the excisional procedure, the rate of HPV viral clearance was 84.6%, and the rate of HPV persistence was 15.4%.

A multinomial logistic regression model was estimated to assess the influence of predictors on the final outcome, with the following three categories: “viral clearance after conization”, “viral clearance after conization and vaccination”, and “persistence”. For the dependent variable, the category “persistence” was the reference group. The following predictors were included: age, CIN 1, CIN 2, CIN 3 (serving as the reference category), HPV 16/18 positivity (serving as the reference category), and positivity for other hrHPV genotypes. The global significance of each predictor was assessed using Likelihood Ratio Tests, and the model’s overall explanatory power was evaluated using Pseudo R-square, specifically the Nagelkerke and Cox and Snell coefficients. The results of the estimated logistic regression are presented in Table 3.

The estimates show that, compared with the “persistence” category, no statistically significant factors were identified for the “viral clearance after conization” category. For the “viral clearance after vaccination” group, the “HPV” result variable had a significant influence on the dependent variable, with a *p* value of 0.001. Women with CIN 2 lesions have a 94.8% lower chance of viral clearance after vaccination than those in the CIN 3 group, making them more likely to remain in the persistence group. The variables “age” and “HPV 16/18” are at the limit of significance, with a *p* value of 0.061, indicating borderline significance. The results show that, with increasing age, the chances of healing after vaccination decrease slightly (by approximately 9% for each additional year). Regarding the “HPV 16/18” variable, the results show that patients who did not present the 16/18 strains have a 5.5 times higher chance of healing after vaccination than those who were positive. The absence of hrHPV 16 and 18 genotypes increases the chances of viral clearance after vaccination, but these chances decrease with increasing age.

## 4. Discussion

According to the European Cancer Inequalities Registry, cervical cancer mortality decreased significantly in Romania by 25% between 2011 and 2021, compared to a 16% decrease across Europe; despite this, however, it remained three times as high as the European average in 2021, with 12 deaths per 100,000 people in Romania compared to 4 per 100,000 across Europe. The main contributing factors to this situation are the following: low screening coverage; limited HPV vaccination (lack of confidence in the benefits of vaccination or financial difficulties); and healthcare disparities (socio-economic inequalities) [30,31,32,33,34,35,36,37,38].

There are four ongoing trials with unpublished results that we hope will shed some light as to what the actual benefits of HPV vaccination are, as related to surgical excisional procedures, as well as the best moment for vaccine administration, to maximize favorable outcomes in women with cervical dysplasia (Novel trial from Finland and Sweden NCT03979014; HOPE9 trial from Italy NCT03848039; VACCIN study from Netherlands NL 7938 Dutch Trial Register and COVENANT trial on seropositive women from South Africa NCT03284866) [10]. The incidence of HPV infection exceeds 70% in sexually active women, with considerable regression rates within a 2-year timeframe. The immune system fails in clearing the infection in a small number of cases (10–20%)—cases that end up developing SIL. Currently, there is no consensus regarding the risk factors for HPV infection persistence, nor are there adequate therapeutic strategies [39,40].

There is growing evidence that vaccination around the time of excisional procedures for cervical dysplasia, ranging from 1 month before to 3–12 months after, may confer benefit [9,10,16,20,23]. However, other studies report no statistically significant reduction in these risks, and conflicting results create uncertainty in the management of recurrences and persistent disease [3,10,24,39,41,42,43,44,45,46,47]. The nonavalent HPV vaccine, while highly effective at preventing new infections, does not clear pre-existing HPV infections in women who are already HPV-positive at the time of vaccination. Based on our results, vaccination timing appears to be critical, as sufficient time is needed for cell-mediated immunity to develop and to prevent HPV from infecting the remaining normal tissue after conization. All women vaccinated before conization achieved viral clearance; 94.9% of those vaccinated at the time of conization/LLETZ and 68.6% of those vaccinated after the excisional procedure became HPV-negative. However, there was no significant difference in the final outcome between unvaccinated and vaccinated women, with a *p*-value of 0.005. Post-excisional vaccination might have limited effect because several months after the surgical procedure, scar tissue creates a less favorable environment for an optimal vaccine-induced immune response compared with vaccination in tissue that is still actively regenerating [8,13,14,16,18,22,23,25,26].

This result underscores the importance of excisional procedures in enhancing local immunity and creating an HPV-free environment, as stated by Ghelardi et al. in the above-mentioned trial [16]. Our results indicate that HPV persistence and clearance appear to be influenced by the timing of vaccination, with women vaccinated before conization or within one year after conization experiencing higher rates of viral clearance than those vaccinated after conization. The difference in viral clearance outcomes reaches statistical significance at the end of our two-year follow-up period. Among the 11 HPV-positive women who chose to be vaccinated after the 6-month follow-up analysis, high rates of HPV clearance were still observed. These results may reflect that early post-conization periods capture residual or persistent infections more readily, as the excisional procedure disrupts the local environment and vaccine-induced immunity requires time to fully develop and clear HPV infection. However, the 2-year time frame may also be associated with higher spontaneous regression rates, and the cumulative effect of the procedure itself (conization/LLETZ) could dilute the association, as many cases achieve clearance regardless of the initial cytology.

When analyzing CIN grade at the moment of conization, we detected 11.2% (9/80) of cases with CIN I lesions and 39% (71/182) of cases with CIN 2+ lesions in our vaccinated group; among our unvaccinated group, 20.1% (28/139) of cases had CIN I lesions, and 61% (111/182) of cases had CIN 2+ lesions. The higher prevalence of CIN 1 lesions in unvaccinated women may reflect differences in referral patterns or lesion regression potential, although this did not significantly impact overall outcomes; this, nevertheless, underscores the need for cautious interpretation. The higher proportion of CIN 1 lesions in our unvaccinated group might have been subject to selection bias. Despite the higher proportion of low-grade CIN 1 lesions observed in the unvaccinated group, the final analysis revealed the statistically significant impact of vaccination on reduction in more severe cervical lesions, further highlighting its beneficial effects.

The rates of HPV-negative ASC-H and HSIL observed in our study are consistent with those reported in the literature, which typically range from 5 to 8%. Specifically, our findings are consistent with the 6.84% rate of HPV-negative ASC-H/HSIL lesions reported by Liu et al. [48]. However, our analysis detected almost twice the rates of HPV-negative ASC-H and HSIL as those in the literature, likely due to sampling variability, selection bias, or assay sensitivity [48]. This aspect is also concordant to the fact that women with higher grade lesions are subjected to invasive procedures like conization/LLETZ, so our inclusion and exclusion criteria might have led to this discrepancy. But as a consequence, the higher rate of HPV negative ASC-H and HSIL may overestimate vaccination benefits in HPV-positive subsets and warrants further investigation in larger studies.

Our multinomial logistic regression results are consistent with the literature, underscoring the importance of age and the HPV genotypes 16 and 18 in achieving viral clearance. Although the pseudo-R-square values were modest (Cox and Snell R^2^ = 0.095, Nagelkerke R^2^ = 0.131), they indicate that the model accounts for a meaningful portion of the variance in the outcome and are consistent with findings in multifactorial clinical studies where outcomes are influenced by a wide array of biological and environmental variables [49].

A total of 33 women (15.1%) were HPV negative at baseline: 24 in the unvaccinated group and 9 in the vaccinated group. Most HPV-negative women with cervical dysplasia considered the lesion HPV-independent and therefore deemed vaccination unnecessary. Because our primary endpoint was HPV clearance or persistence, their distribution had only a minor influence on our results (*p* value of 0.511), and our main finding was the timing of HPV vaccination synchronous with the excisional procedure. The slight imbalance in their distribution between the HPV-vaccinated and unvaccinated subgroups might only dilute the apparent benefit of vaccination in the unvaccinated. We also added this information to the discussion section.

In this study, we report several noteworthy findings. To our knowledge, this is the first study conducted in Romania evaluating the outcomes of prophylactic, nonavalent HPV vaccination administered for therapeutic purposes at the time of LLETZ/conization in women diagnosed with HPV-related cervical dysplasia. Romania ranks first in Europe for both incidence and mortality from cervical cancer, according to GLOBOCAN 2022, and this is the first study evaluating the importance of the timing of the first vaccine dose [2]. As our results show, younger women are more likely to accept vaccination than older women. We are aware that young age is an advantage for HPV viral clearance, and that our two groups of women were not age-matched, which is a source of bias in our results. Our results reflect not only the general mindset of the Romanian population but also that of the North-East region, which ranks lower in economic development and educational level. According to data from the National Institute of Statistics, the HPV vaccination rate in Iasi County was only 5% in 2024, with 1326 individuals vaccinated out of 29,215 in the 11–45 age group [2,50,51].

In our cohort of patients treated with LLETZ/conization for cervical dysplasia, six women had been vaccinated prior to diagnosis. Although all patients were offered HPV vaccination at the time of scheduling surgery, only 39 women (48.7%) received the first dose before their first follow-up visit (on average, at the 6-month follow-up point). Thirty-five patients were vaccinated only after the first follow-up results, when HPV persistence was confirmed. Although limited in number, this study included data on 11 patients with residual HPV infection or reinfection at the first follow-up, who agreed to initiate vaccination more than 6 months after conization.

Additionally, the HPV testing method used in our study enabled extended genotyping, allowing for the evaluation of vaccine effectiveness for individual HPV types. Most published studies use genotyping tests only capable of identifying a limited range of HPV types, typically types 16, 18, and a few other hrHPV genotypes [26,52]. The 16 hrHPV genotype was detected in 92 of the women included in our study (27 women from the vaccinated group and 67 from our unvaccinated group), and the 18 hrHPV genotype was detected in 27 of the patients included in our study (18 from the unvaccinated group and 2 from the vaccinated group). This study was also one of few in which the nonavalent prophylactic HPV vaccine was exclusively used, administered with therapeutic intent at the time of conization. In 2024, Dvořák et al. published the first cohort study assessing the effectiveness of post-conization vaccination exclusively with the nonavalent HPV vaccine [53]. Due to national vaccination policies, the other two HPV vaccines are no longer available in Romania, and none of the patients enrolled in our study had received any HPV vaccine prior to conization. However, according to Dvořák, whether the nonavalent vaccine enhances effectiveness remains inconclusive. Similarly, our study did not conclusively demonstrate that HPV vaccination reduces CIN2+ recurrence, as only a non-significant 42% reduction was observed, likely due to the limited sample size of women with positive cone margins [48,53].

The decision to perform conization was made in accordance with national guidelines, taking patients’ preferences into account and balancing risks against cancer prevention. Although HPV-negative ASC-US is generally considered low risk, one patient had an unsatisfactory colposcopy with features suggestive of CIN2 (confirmed by pathological examination of the sample) and postcoital bleeding—both of which increase the pretest probability of clinically significant disease. In the clinical context, characterized by elevated patient anxiety and limited feasibility of longitudinal follow-up owing to high population mobility and the absence of comprehensive health insurance coverage, proceeding directly with a diagnostic excisional procedure is a reasoned management strategy. This approach is also justifiable in settings where patient follow-up is unreliable due to socioeconomic and educational barriers, as immediate treatment decreases the risk of loss to follow-up and progression of undetected high-grade lesions.

We presented both HPV status and Pap smear results because our study outcomes and definitions were deliberately designed to capture both virological and cytological endpoints. We acknowledge that this dual approach differs from some studies that rely exclusively on histological findings and confirmation or HPV clearance alone. However, we believe it is clinically justified for the following reasons: although HPV testing is highly sensitive for detecting persistent infection, a proportion of cervical neoplasia can occur in the absence of detectable HPV [54]. Thus, the combined assessment provides a more comprehensive evaluation of post treatment risks in settings where patients frequently miss appointments, and cervical neoplasia screening is still opportunistic.

Our research emerged from our desire to establish the optimal moment for vaccination. The literature indicates that vaccination before conization may lead to favorable outcomes, whereas late vaccination is associated with failure to prevent reinfection [47]—findings that are quite similar to ours. Compared to Petráš et al.’s recent research, our cohort was homogenous, all vaccinated women had the nonavalent vaccine administered, all HPV-positive women had HPV genotyping performed, and we had access to data about the completion of the vaccination schedule and the moment of administration related to the excisional procedures [19].

This study, however, has several limitations, listed as follows: the small sample size; the single-center design, which reduces the generalizability of our results; selection bias and the lack of randomization; age disparities between our subgroups, which influence outcomes; the lack of data on several constitutional variables (we had no data on whether the women’s partners received prophylactic vaccination, which likely explains our high recurrence rate and parity); the possibly insufficient mean length of follow-up (two years) compared with other timeframes reported in the literature [27,47]; and incomplete data on socio-behavioral parameters, which led to the exclusion of some participants from our analysis. Moreover, because outcome assessments were not blinded to vaccination status, this represents an additional source of bias inherent to the retrospective study design, in which vaccination status was part of the medical record and could not be blinded post hoc. If the lack of blinded vaccination status may introduce detection bias, particularly for subjective endpoints such as cytological interpretation, HPV testing is less susceptible to such bias. Thus, our findings need to be carefully interpreted in light of these limitations.

Nonetheless, the strengths of our research include the following: all data were obtained from a setting with a high incidence of CIN lesions and cervical cancer, suboptimal vaccination rates, and a substantial HPV-related disease burden. The vaccine used was Gardasil^®^ (nonavalent) in all women included in our study, and we clearly pinpointed the vaccination date relative to the excisional procedures. Our population included a variety of ages; thus, our results can be projected across all age clusters.

## 5. Conclusions

Adjuvant prophylactic HPV vaccination in the context of surgical treatment for cervical precancerous lesions is significantly associated with a reduced risk of lesion recurrence and HPV persistence or reinfection when administered before or at the time of excisional procedures. HPV vaccination may be considered an adjuvant approach, particularly in patients undergoing conization for high-grade cervical lesions. To support a future vaccination strategy for these cases, further studies with larger randomized patient cohorts are necessary.

## Figures and Tables

**Table 1:** HPV prevalence among the 219 women in our study at the end of follow-up (after 2 years).

	Vaccination		*p*-Value	Moment of Vaccination			*p*-Value
	Yes	No		Before Conization	Together with Conization	After Conization	
Persistent	13 (16.2%)	20 (14.4%)	0.845 *	0(0%)	2 (5.1%)	11 (31.4%)	0.005 **
Viral clearance	67 (83.8%)	119 (85.6%)		6 (100%)	37 (94.9%)	24 (68.6%)	
Total	80 (36.53%)	139 (63.47%)		6 (7.5%)	39 (48.7%)	35 (43.8%)	

* Chi-Square Test. ** Fisher’s Exact Test.

**Table 2:** Pap smear results of the 219 women included in our study at six months, one year, and two years post-conization/LLETZ follow-up.

		Vaccination					
		Yes			No		
		Persistent	Negative Pap Smear	*p*-Value	Persistent	Negative Pap Smear	*p*-Value
Control 6 months	ASCUS	1 (7.7%)	2 (3%)	0.002 *	3 (15%)	8 (6.7%)	0.014 *
	HSIL	2 (15.4%)	0(0%)		1 (5%)	0(0%)	
	LSIL	2 (15.4%)	1 (1.5%)		2 (10%)	3 (2.5%)	
	NILM	8 (61.5%)	64 (95.5%)		14 (70%)	108 (90.8%)	
Total		13 (100%)	67 (100%)		20 (100%)	119 (100%)	
Control 1 year	ASCUS	4 (30.8%)	0(0%)	<0.0001 *	4 (20%)	1 (0.8%)	<0.0001 *
	LSIL	3 (23.1%)	3 (4.5%)		3 (15%)	0(0%)	
	NILM	6 (46.1%)	64 (95.5%)		13 (65%)	118 (99.2%)	
Total		13 (100%)	67 (100%)		20 (100%)	119 (100%)	
Control 2 years	ASCUS	3 (23.1%)	1 (1.5%)	0.001	2 (10%)	1 (0.8%)	0.054 *
	LSIL	2 (15.4%)	1 (1.5%)				
	NILM	8 (61.5%)	65 (97%)		18 (90%)	118 (99.2%)	
Total		13 (100%)	67 (100%)		20 (100%)	119 (100%)	

* Fisher’s Exact Test.

**Table 3:** The multinomial logistic regression model for the final outcome.

Dependent Variable	Predictors	Coefficients	OR	95% CI *	*p*-Value
Viral clearance after conization	Constant	1.081 (0.911)			0.236
	Age	0.111 (0.025)	1.011	[0.963, 1.061]	0.658
	Results HPV_CIN1	0.180 (0.642)	1.197	[0.340, 4.213]	0.780
	Results HPV_CIN2	0.371 (0.529)	1.449	[0.514, 4.091]	0.483
	HPV 16/18 (negative)	−0.101 (0.441)	0.904	[0.381, 2.146]	0.820
Viral clearance after vaccination	Constant	2.723 (1.681)			0.105
	Age	−0.096 (0.054)	0.908	[0.817, 1.009]	0.074
	Results HPV_CIN1	−2.274 (1.257)	0.103	[0.009, 1.211]	0.071
	Results HPV_CIN2	−2.963 (0.955)	0.052	[0.008, 0.336]	0.002
	HPV 16/18 (negative)	1.715 (0.895)	5.559	[0.962, 32.105]	0.055

* The reference category for the dependent variable is the category Persistent. For the independent variable Results HPV, the reference category is CIN3 and for HPV 16/18 status the reference category is the positive group. Odds Ratios (OR) and 95% Confidence Intervals (CI) were calculated for each category.

## Data Availability

The original contributions presented in this study are included in the article/ Supplementary Material. Further inquiries can be directed to the corresponding author.

## Supplementary Materials

The following supporting information can be downloaded at: https://www.mdpi.com/article/10.3390/jcm15093424/s1, Figure S1: Flowchart of women with CIN I lesions included in our study; Figure S2: Flowchart of women with CIN 1+ lesions included in our study; Figure S3: Flowchart of women with CIN 2+ lesions included in our study.

## Abbreviations

The following abbreviations are used in this manuscript:

HPV	Human Papillomavirus
CIN	Cervical intraepithelial neoplasia
LEEP	Loop electrosurgical excision procedure
LLETZ	Large loop excision of the transformation zone
hr	High-risk
lr	Low-risk
ASCUS	Atypical squamous cells of undetermined significance
LSIL	Low-grade squamous intraepithelial lesion
HSIL	High-grade squamous intraepithelial lesion
NILM	Negative for intraepithelial lesions or malignancy
ASC-H	Atypical squamous cells, cannot exclude high-grade squamous intraepithelial lesion
