# Supplementary material for: Integrating Adjuvant HPV Vaccination into Cervical Dysplasia Management After LLETZ/Conization
Source: J Clin Med. 2026 Apr 30;15(9):3424. doi: 10.3390/jcm15093424 (PMC13164224; doi:10.3390/jcm15093424)
Supplement: Supplementary file 1 [file jcm-15-03424-s001.zip › jcm-4186934-supplementary.pdf]

Journal of
Clinical Medicine

MDPI

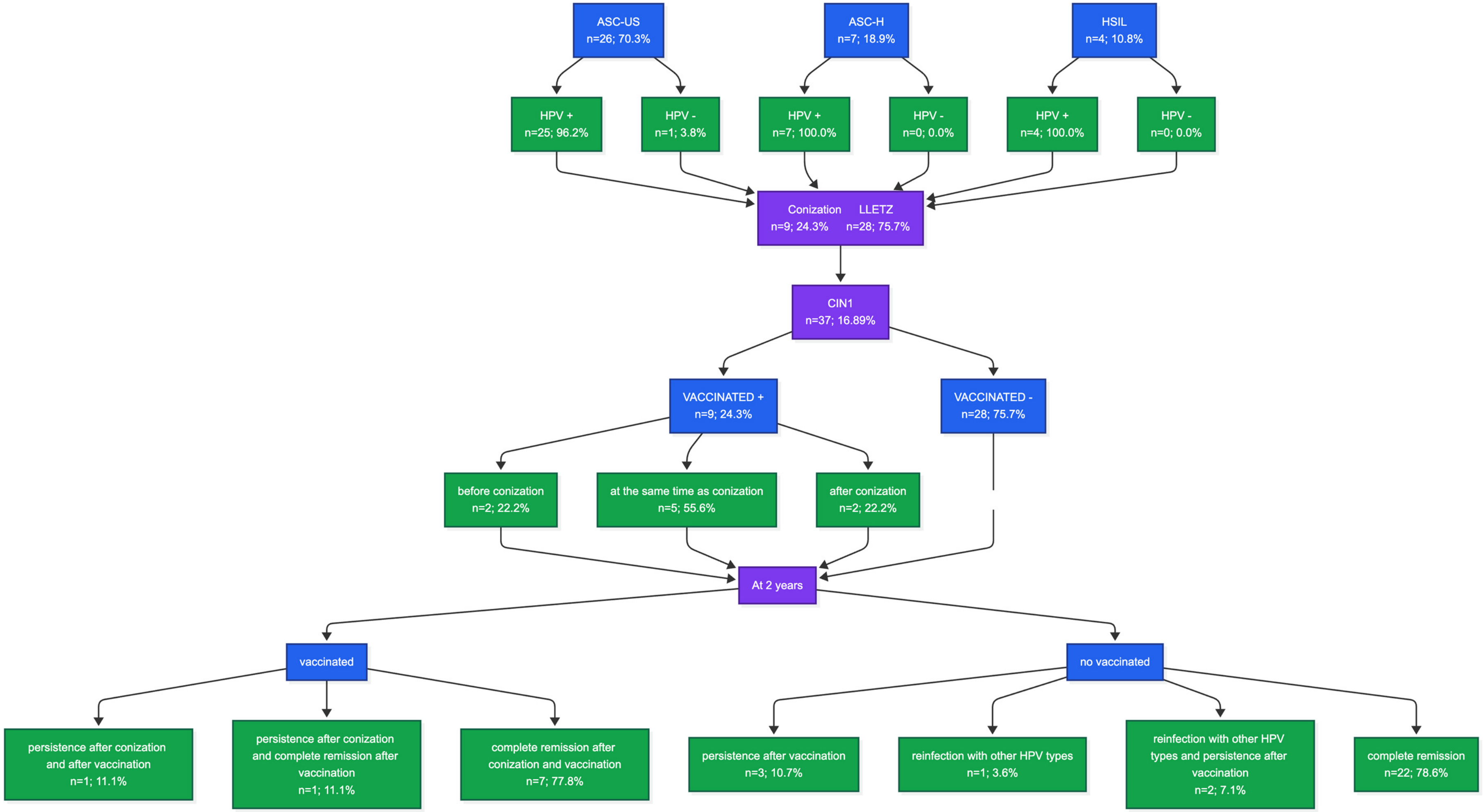

Figure S1. Flowchart of women with CIN I lesions included in our study.

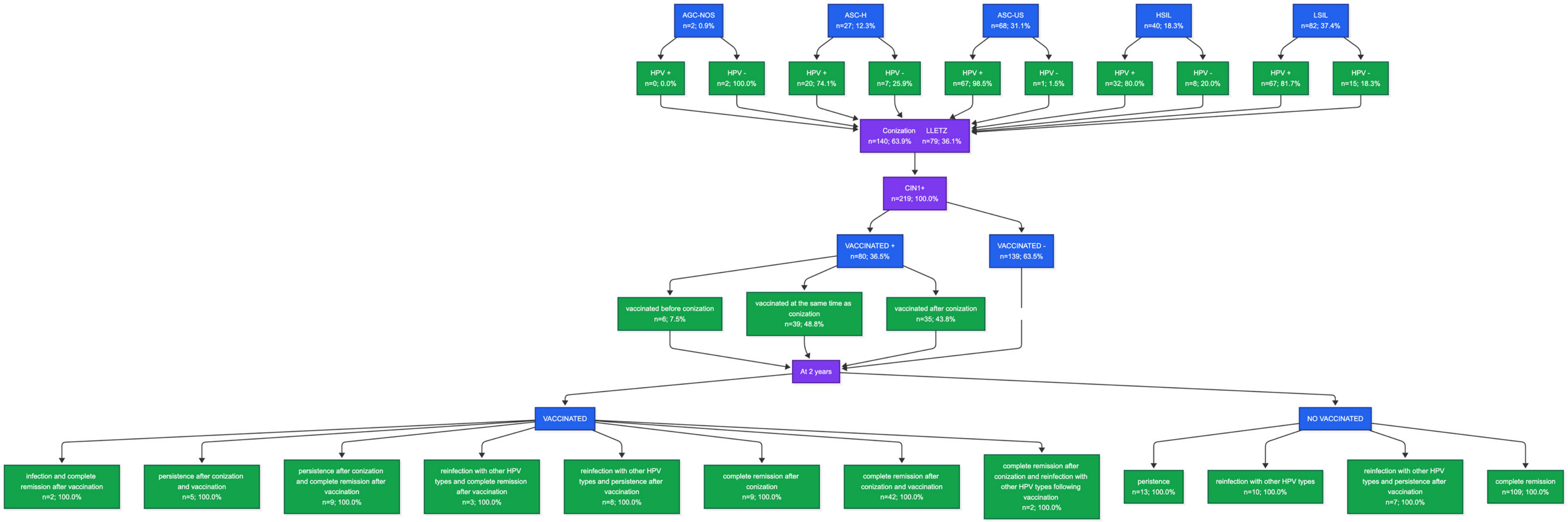

Figure S2. Flowchart of women with CIN 1+ lesions included in our study.

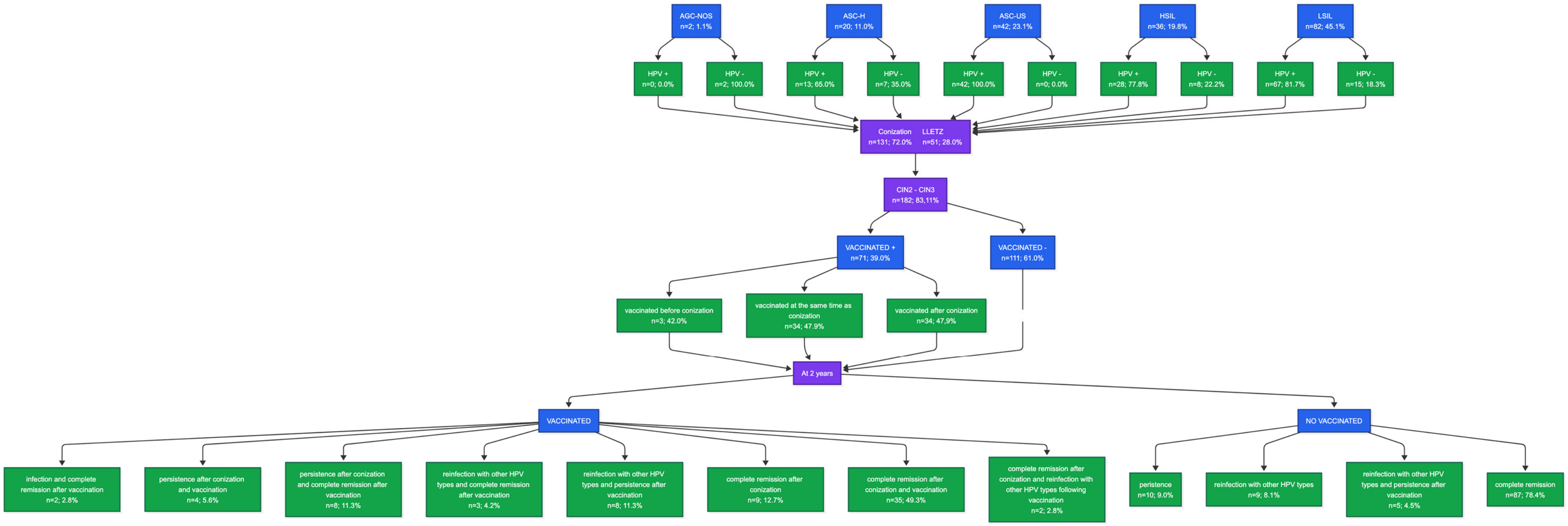

Figure S3. Flowchart of women with CIN 2+ lesions included in our study.
